# Hatchery tanks induce intense reduction in microbiota diversity associated with gills and guts of two endemic species of the São Francisco River

**DOI:** 10.3389/fmicb.2022.966436

**Published:** 2022-12-02

**Authors:** Maria Rosilene Alves Damasceno, Camila Gracyelle de Carvalho Lemes, Lucélia Sandra Silva Barbosa Braga, Polyana Cristine Tizioto, Horácio Montenegro, Marcela Paduan, Josielda Gomes Pereira, Isabella Ferreira Cordeiro, Lorrana Cachuite Mendes Rocha, Sibele Aryadne da Silva, Angélica Bianchini Sanchez, Wanderson Geraldo Lima, Gabriel Menezes Yazbeck, Leandro Marcio Moreira, Camila Carrião Machado Garcia

**Affiliations:** ^1^Programa de Pós-Graduação em Ciências Biológicas, Núcleo de Pesquisas em Ciências Biológicas, Universidade Federal de Ouro Preto, Ouro Preto, Brazil; ^2^Programa de Pós-Graduação em Biotecnologia, Núcleo de Pesquisas em Ciências Biológicas, Universidade Federal de Ouro Preto, Ouro Preto, Brazil; ^3^Instituto Federal de Educação, Ciência e Tecnologia do Norte de Minas Gerais, Januária, Brazil; ^4^NGS Soluções Genômicas, Piracicaba, Brazil; ^5^DEZOO, Universidade Federal de São João del-Rei, São João del-Rei, Brazil; ^6^Departamento de Ciências Biológicas, Instituto de Ciências Exatas e Biológicas, Universidade Federal de Ouro Preto, Ouro Preto, Brazil

**Keywords:** São Francisco River, Neotropical fish, microbiome, next-generation sequencing, aquaculture, conservation

## Abstract

The São Francisco River (SFR), one of the main Brazilian rivers, has suffered cumulative anthropogenic impacts, leading to ever-decreasing fish stocks and environmental, economic, and social consequences. *Rhinelepis aspera* and *Prochilodus argenteus* are medium-sized, bottom-feeding, and rheophilic fishes from the SFR that suffer from these actions. Both species are targeted for spawning and restocking operations due to their relevance in artisanal fisheries, commercial activities, and conservation concerns. Using high-throughput sequencing of the 16S rRNA gene, we characterized the microbiome present in the gills and guts of these species recruited from an impacted SFR region and hatchery tanks (HT). Our results showed that bacterial diversity from the gill and gut at the genera level in both fish species from HT is 87% smaller than in species from the SFR. Furthermore, only 15 and 29% of bacterial genera are shared between gills and guts in *R*. *aspera* and *P. argenteus* from SFR, respectively, showing an intimate relationship between functional differences in organs. In both species from SFR, pathogenic, xenobiont-degrading, and cyanotoxin-producer bacterial genera were found, indicating the critical pollution scenario in which the river finds itself. This study allowed us to conclude that the conditions imposed on fish in the HT act as important modulators of microbial diversity in the analyzed tissues. It also raises questions regarding the effects of these conditions on hatchery spawn fish and their suitability for restocking activities, aggravated by the narrow genetic diversity associated with such freshwater systems.

## Introduction

**Figure 1:**
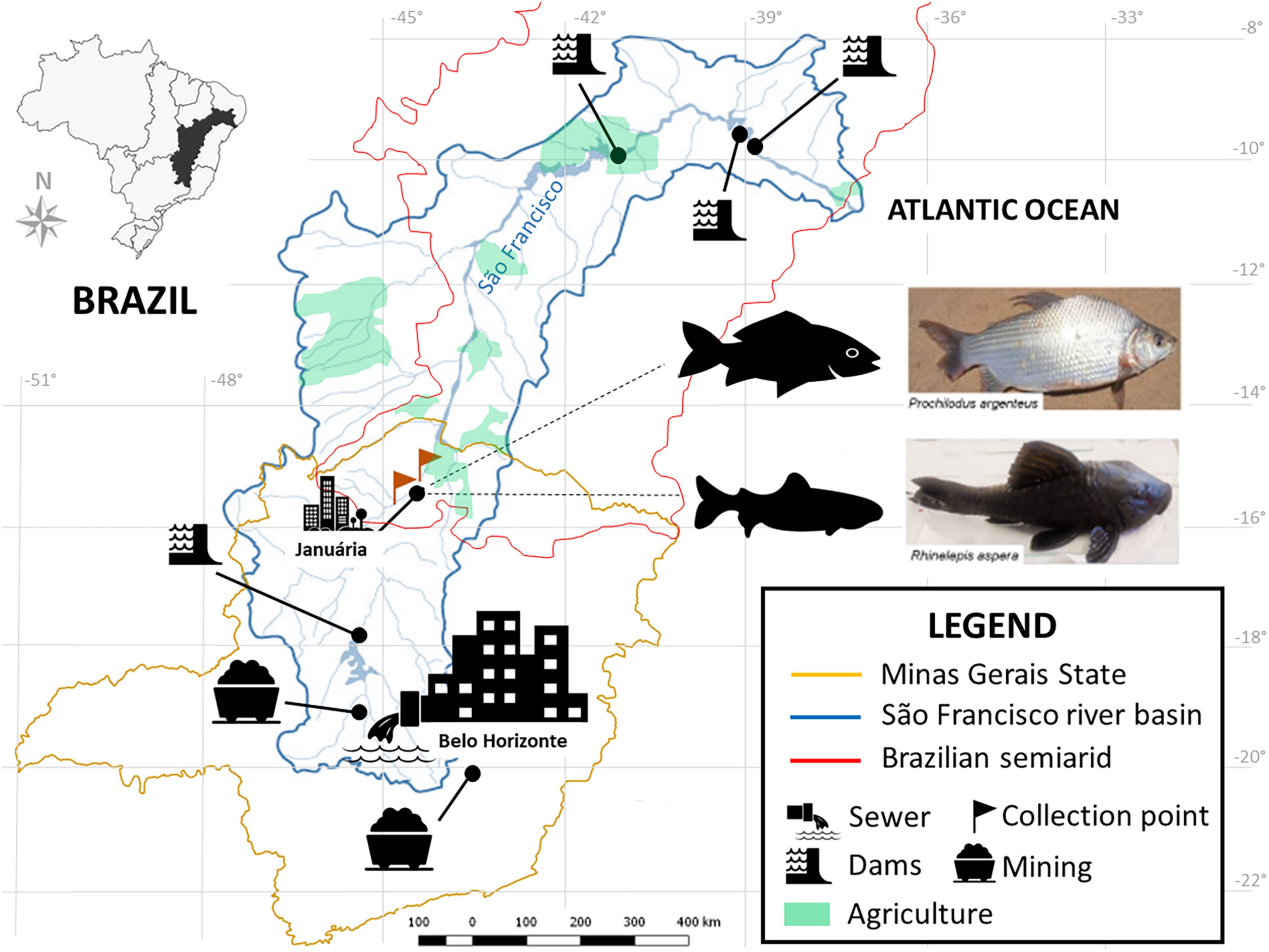
Location of the SFR Basin, highlighting the region of collection and the species of ichthyofauna studied. Fish collection site in the middle SFR region, upstream and downstream of the city of Januária, North of Minas Gerais. Brazil. Google. The map was obtained from the Pristine Institute: digital geoenvironmental atlas. WebGis (Web Geographical Information System) with “free access to the environmental database.” Available at <https://institutopristino.org.br/atlas/>. Access on 25/Jun/2020.

Human impacts on fish biodiversity affect more than 50% of the world’s freshwater systems (Su et al., 2021). The Neotropical region harbors over 6.200 named freshwater fish species, making it the most diverse continental vertebrate fauna on the planet (Albert et al., 2020). The São Francisco River (SFR) is the fifth-largest river in South America, more than 2,700 km in length, enclosed in a 639.219 km^2^ eponymous basin, and it constitutes the most extensive river residing entirely inside Brazilian territory (Sun et al., 2016) (Figure 1). It cuts through two major biomes, the Brazilian savannah, Cerrado, and the Caatinga, allowing for agricultural operations around it in otherwise dry regions, as well as inland artisanal and commercial fisheries (Holanda et al., 2009; Maneta et al., 2009; Torres et al., 2012).

The SFR is widely afflicted by the cumulative effects of several anthropogenic impact sources, such as the introduction of invasive species (Linares et al., 2019); suppression of riverine vegetation (Holanda et al., 2009); mining and damming for hydropower generation activities (Nascimento and Becker, 2010), and urban and rural pollution (Ribeiro et al., 2012) (Figure 1). In addition, during the last decade, unstable hydrological regimes in dry regions (Otto et al., 2015; Getirana, 2016; Gudmundsson et al., 2021) and overfishing have contributed to ever-decreasing fish stocks (Barbosa et al., 2017; Figueiredo, 2018). Thus, sustainability in the SFR is threatened, jeopardizing the livelihood of its communities of part-time and full-time fishing folk (Gutberlet et al., 2007; Alves and Leal, 2010; Barbosa et al., 2017).

Fish spawning and stocking operations have been set as hatchery tanks (HT) for fish farming, employed to produce angling opportunities, commercial sales, and to counteract environmental pressures (Arantes et al., 2011). However, its efficiency remains to be shown, especially if disconnected from broader policy actions to address the root causes of fish stock decline in the SFR (Araki and Schmid, 2010; González-Wangüemert et al., 2012; Klinger and Naylor, 2012; Savary et al., 2017). In addition, there are concerns related to the potentially deleterious effects of genetic diversity impoverishment in spawned fish and sanitary hazards observed in hatcheries (González-Wangüemert et al., 2012; Carmo et al., 2015; Benson et al., 2016; Pavlova et al., 2017; Savary et al., 2017; FAO, 2020).

Environmental factors, both biotic and abiotic, modulate the assemblage of microorganisms (microbiome) associated with organisms and their different morphophysiological features (Sullam et al., 2012; Duarte et al., 2014; Ringo et al., 2016; Sylvain et al., 2016; Guivier et al., 2017).

Few studies are addressing the morphophysiological changes caused to Neotropical fish by chronic exposure to environmental toxins in their natural habitat (Paschoalini and Bazzoli, 2021). Also, the presented data are not robust enough to link aquatic contamination to mortality risks of the ichthyofauna species (Molbert et al., 2021). However, there are reports of histological and molecular changes in the liver, spleen, and gills of *P. argenteus* recruited from regions impacted by heavy metals in the SFR basin. Among them, a significant increase in melanomacrophage centers in the liver and spleen, a high incidence of fibrosis in the spleen, alterations in the number and morphology of germ cells, and also endocrine and reproductive dysfunctions, with a negative effect on the gonadal development of females (Paschoalini et al., 2019). In the gills, increased inflammatory foci, hyperplasia, edema, and lamellar fusion were reported (Arantes et al., 2011; Procópio et al., 2014). Additionally, toxicogenetic biomarkers positively correlated the increased frequency of micronuclei in erythrocytes (Shirmohammadi et al., 2018; Delcorso et al., 2020) with the speed of telomere shortening (Molbert et al., 2021).

Changes in microbiome composition have been implicated in increased disease occurrence, population size decline, and other adverse effects in animals (Sylvain et al., 2016; Pękala-Safińska, 2018). Also, the microbial composition is related to vital metabolic processes in the host (Izvekova et al., 2007; Lowrey et al., 2015; Liu et al., 2016; Sylvain et al., 2016; van Kessel et al., 2016).

Significant advances toward a complete understanding of microbial-environmental interactions have been strengthened by the development of culture-free methods in microbiology, such as high-throughput sequencing of specific genes or the assembly of complete microbial genomes (Faust and Raes, 2012; Blainey, 2013; van Dijk et al., 2014), which allows the identification of a myriad of prokaryotic species, more significant in scope than with the few species eventually detected by growth on culture media.

The importance of fish-microbiome interaction, its relationship to aquaculture, and its role in survival and performance has been acknowledged in this century (van Kessel et al., 2011; Ringo et al., 2016; Tarnecki et al., 2016; Perry et al., 2020). Characterizing microbiome composition could help craft ways to facilitate the functional enhancement of nutritional, immunologic, and other beneficial traits in reared fish (Perry et al., 2020). Studies involving fish have provided insights into the importance of microbiome associated with the skin, gills, and gut, allowing for the characterization of factors influencing microbial diversity in both wild and reared species; at least 145 fish gut microbiomes have been analyzed to date (Roeselers et al., 2011; Sylvain et al., 2016, 2019; Tarnecki et al., 2016; Uren Webster et al., 2018; Krotman et al., 2020; Nolorbe-Payahua et al., 2020; Perry et al., 2020; Riiser et al., 2020). Nevertheless, most of these studies have been performed on economically relevant fish such as *Cyprinus carpio* (van Kessel et al., 2016), *Ctenopharyngodon idellus* (Wu et al., 2012), *Salmo salar* (Gajardo et al., 2016; Schmidt et al., 2016), *Oreochromis niloticus* (Yu et al., 2019; Xia et al., 2020), *Oncorhynchus mykiss* (Lowrey et al., 2015), *Colossoma macropomum* (Sylvain et al., 2016), *Arapaimas gigas* (Ramírez et al., 2018), *Seriola lalandi* (Legrand et al., 2018) and *Ictalurus punctatus* (Larsen et al., 2014). Thus, there is still a relatively low number of studies focusing on microbiomes in wild living fish. Studies involving fish microbiomes from the SFR are still scarce (Silva et al., 2005; Makino et al., 2012; Santos et al., 2014; Da Silva et al., 2020). Even though important, they are traditionally limited by culture-dependent methods.

So far, most metagenome research has focused on the fish gut microbiota. These studies have shown that diet and physicochemical variations of water are strong modulators of microbial diversity, leading to metabolic dysbiosis, increased susceptibility to infections, and reduced environmental adaptability (Ning et al., 2020). Such alterations were observed in *Gambusia affinis* and *Danio rerio* exposed to Benzo(a)pyrene, which induced intestinal dysbiosis and the increased expression of genes related to the inflammatory response (Xie et al., 2020). Exposure of *D. rerio* to titanium dioxide nanoparticles and Bisphenol A induced an increase in the *Lawsonia* genera, a pathogen of the gastrointestinal tract of animals, and a decrease in *Hyphomicrobium* associated with denitrification metabolism (Chen et al., 2018). Reported as polycyclic aromatic hydrocarbons (PAHs) degraders, the bacterial genera *Novosphingobium*, *Sphingobium*, and *Sphingomonas* were found in the intestinal microbiota of *Gadus morhua* collected in impacted environments by oil contamination while absent in unimpacted environments (Walter et al., 2019).

This work aimed to characterize the microbiomes present in the gills and guts of two relevant bottom-feeding species from the SFR (*R. aspera* and *P. argenteus*) and to establish a comparison with the same species from an HT for fish farming intended for restocking activities through a culture-free approach through high-throughput sequencing of the 16S rRNA gene.

In this study, we hypothesized that benthic fish, iliophagous detritivores, which differ in behavior in their natural habitat, being one a long-distance migrator (*P. argenteus*) and the other a sedentary one (*R. aspera*), are exposed to different levels of environmental stress and physicochemical gradients of water, differentially modulating the structure and diversity of the intestinal and gill microbiome of these species. Additionally, this study was the first to characterize the microbiomes of these two ichthyic species recruited in the SFR, providing important insights into the effects of confinement on the structure and diversity of the investigated microbiomes, pointing out vulnerabilities associated with commercial aquaculture and restocking programs, and aiming at the restoration of natural ichthyofauna stocks.

## Methodology

**Figure 2:**
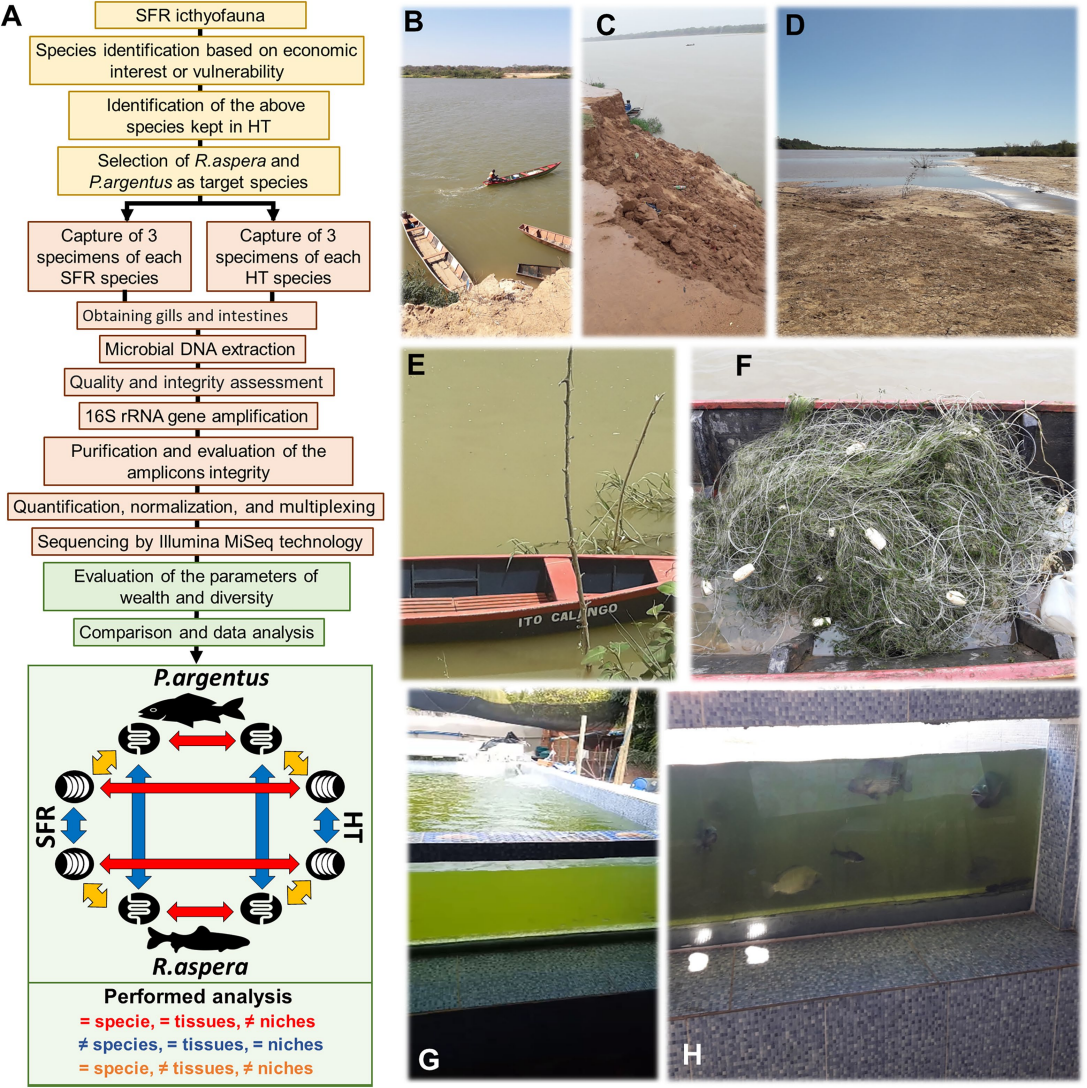
**(A)** Experimental design. The planning steps are on a yellow background, the collection and processing steps on a salmon background, and the steps to obtain results and data analysis on a green background. Photos that, respectively, denote degradation of riparian forests **(B)**, which causes landslides **(C)** that in turn contribute to silting up of the SFR bed **(D)**. **(E)** Evident eutrophication of the SFR waters at the time of the first collection is best proven when trawls are used **(F)**. **(G,H)** HT structure where they were collected.

The complete methodological workflow of this study is presented in Figure 2A. Two species (*R. aspera and P. argenteus*) were selected based on relevance in fisheries, drastic stock reduction, and vulnerability. In addition, as bottom feeders, both species constitute adequate water column indicators and sediment contamination (Camargo et al., 2009; Islam et al., 2015; Paschoalini et al., 2019).

### Morphophysiological and behavioral characterization of species

*Rhinelepis aspera* (Spix and Agassiz, 1829) is a medium-sized, stream-dwelling, melanic, armored catfish locally known as *acari* or *cascudo preto* (Siluriformes order, the Loricariidae family (Britski et al., 1988; Buckup et al., 2007). It is among the single largest species (> 4 kg) within this family, which, in turn, is characterized by the presence of thick bony plates covering the skin, ventral suckermouth, and round eye’s iris (Covain and Fisch-Muller, 2007). The specie *R. aspera* exhibits obligatory male parental care of young adults and lacks long migratory movements associated with reproduction (wet season), which is common in large Neotropical catfish (Suzuki et al., 2000; Sato et al., 2003). The International Union for Conservation of Nature does not assess its conservation status (International Union for Conservation of Nature and Natural Resources –IUCN, 2022). Still, it was deemed a species endangered at the end of the 20th century, and it has been reported as rare in the main body of the SFR basin, although abundant in its main tributary, the Paracatu River (Sato et al., 1998). It shows a specialized mouth, adapted to its iliophagous lifestyle, being an important organic matter and nutrient recycling player (Roxo et al., 2019). This fish is highly adaptable to lentic environments, and its high fecundity makes it a natural target for fish farming initiatives in the SFR region.

The specie *P. argenteus*, Spix and Agassiz, 1829, is one of the two species of the *Prochilodus* genera (Characiformes order, Prochilodontidae family (Britski et al., 1988; Castro and Vari, 2004) occurring in the SFR basin. This region could have served as a dispersal departure point for the evolutionary radiation of the five species in southeastern Brazil (Santos et al., 2021). *P. argenteus* is the largest species from its family (Sato et al., 2003) and is popularly known as *zulega*, *curimatã-pacu*, *curimba,* or *papa-terra* (Perini et al., 2021). It can reach approximately 45 cm in length, up to 15 kg, and it is one of the largest and most prominent species for artisanal and commercial fisheries in the SFR (Godinho and Godinho, 2003; Sato et al., 2005; Godinho et al., 2017). It is a typical potamodromous migratory Neotropical fish that performs long upstream movements in the wet season (Sato et al., 2003, 2005), with high tolerance and suitability for aquaculture (Almeida et al., 2015).

### Collection sites and experimental design

This study was conducted in the middle portion of the SFR, immediately upstream and downstream of the city of Januária (S 15°29.576’ W 044°21°0.069′), in the north of the Minas Gerais estate, Brazil (Figure 1). It is a major regional center, with a population of approximately 65.463 and a human development index of 0.658, with only 37.2% of households served by sewage (IBGE, 2010).

*Rhinelepis aspera* and *P. argenteus* specimens were sampled from the SFR (Figures 2B–G) and an HT from Januária (S 15°28′ 48.94752” W 044°21′42.50952″). This tank was built in tiled masonry above the ground, supplied with water from the river after being treated by the Companhia de Saneamento de Minas Gerais (COPASA).

The Instituto Mineiro de Gestão das Águas em Minas Gerais (IGAM, 2018) has classified the water from the sampling point as Class II, with several diagnostic features above the limits established by Brazilian legislation (BRASIL, 2005) like dissolved aluminum, total manganese, lead, total solids, turbidity, biochemical oxygen demand (BOD), and cyanobacteria density (visually confirmed during sampling; Supplementary Table 1 and Figures 2E,F).

Three fish were collected from each species in the SFR and HT, totaling 12 individuals. Fish were trapped with a dragnet (Figure 2F) by professional fishers from the Associação de Pescadores de Januária in July 2018. Two male and one female specimen of the fish were taken alive to the Laboratório de Zoologia do Instituto Federal de Educação, Ciência e Tecnologia do Norte de Minas Gerais, campus Januária. The fish identification was confirmed according to Britski et al. (1988). One voucher specimen for each species was stored in the laboratory collection mentioned before. The fishes were euthanized to remove the medial portions of the gills and guts using sterile instrumentation under a laminar flow hood.

### Total DNA extraction

For associated microbiome DNA extraction, two samples from each fish were collected: gills (G) and medium gut (I) from each species *P. argenteus* (Pa) and *R. aspera* (Ra), from SFR or HT (TQ), totaling 24 samples (Table 1). According to the manufacturer’s guidelines, small sections of approximately 3 g of gill and gut of each animal were used for DNA extraction using the NucleoSpin™ Tissue (Macherey-Nagel, Germany) commercial kit followed by NanoDrop™ (Thermo Fisher Scientific, USA) quantification at 260 nm. In addition, DNA integrity was verified using 0.8% agarose gel electrophoresis.

**Table 1:** Number and characteristics of samples obtained from *R. aspera* and *P. argenteus* collected in the SFR and HT.

Samples	SeqCode	Species	Organs	Environment	#Reads
1	1*Ra*IHT	*R. aspera*	I	HT	162,102
2	1*Ra*GHT	*R. aspera*	G	HT	170,706
3	2*Ra*IHT	*R. aspera*	I	HT	158,763
4	2*Ra*GHT	*R. aspera*	G	HT	182,837
5	3*Ra*IHT	*R. aspera*	I	HT	137,448
6	3*Ra*GHT	*R. aspera*	G	HT	150,649
7	1*Pa*IHT	*P. argenteus*	I	HT	132,940
8	1*Pa*GHT	*P. argenteus*	G	HT	160,301
9	2*Pa*IHT	*P. argenteus*	I	HT	154,155
10	2*Pa*GHT	*P. argenteus*	G	HT	155,418
11	3*Pa*IHT	*P. argenteus*	I	HT	133,581
12	3*Pa*GHT	*P. argenteus*	G	HT	153,282
13	1*Ra*ISFR	*R. aspera*	I	SFR	169,824
14	1*Ra*GSFR	*R. aspera*	G	SFR	160,309
15	1*Pa*ISFR	*P. argenteus*	I	SFR	119,957
16	1*Pa*GSFR	*P. argenteus*	G	SFR	183,156
17	2*Pa*ISFR	*P. argenteus*	I	SFR	151,713
18	2*Pa*GSFR	*P. argenteus*	G	SFR	138,402
19	2*Ra*GSFR	*R. aspera*	G	SFR	150,646
20	2*Ra*ISFR	*R. aspera*	I	SFR	171,690
21	3*Ra*GSFR	*R. aspera*	G	SFR	137,827
22	3*Ra*ISFR	*R. aspera*	I	SFR	172,831
23	3*Pa*GSFR	*P. argenteus*	G	SFR	176,088
24	3*Pa*ISFR	*P. argenteus*	I	SFR	177,679

### Partial amplification of the 16S ribosomal gene

The preparation of the libraries was performed following the recommendations for sequencing on the Illumina platform. Oligonucleotides 341F and 785R (Klindworth et al., 2013) were used, which amplify the V3/V4 region of the 16S ribosomal gene with the included overhang adapter sequences. The sequences of the primers (in uppercase) plus adapters (in lowercase) are 341F: (5’tcgtcggcagcgtcagatgtgtataagagacagCCTACGGGNGGCWGCAG) and 785R: (5’gtctcgtgggctcggagatgtgtataagagacagGACTACHVGGGTATCTAATCC). One μL of DNA was used for the amplification, in addition to 0.5 μl of each primer at 10 μM (0.2 μM final concentration in the reaction, in a final volume of 25 μl), 10 μl of the 2X Ultra Mix PCRBio master mix™ (PCR BioSystems, United Kingdom), topping up with ultrapure water for the final volume of 25 μl. The PCR conditions were: 1 cycle of 95°C for 3 min; 25 cycles: 95°C for 30 s, 55°C for 30 s, 72°C for 30 s; 1 cycle of 72°C for 5 min; keep at 4°C indefinitely. The PCR product was applied on a 1.5% agarose gel and then purified with AMPure XP Beads (Beckmann-Coulter, USA). To connect the adapters, 2.5 μl of the amplification product, 2.5 μl of each index (i5 and i7), 12.5 μl of the 2X Ultra Mix PCRBio master mix™ (PCR BioSystems, USA), and 5 μl of ultra-water were used to adjust the final volume at 25 μl. The PCR conditions for connecting the adapters were: 95°C for 3 min; 8 cycles: 95°C for 30 s, 55°C for 30 s, 72°C for 30 s; 1 cycle of 72°C for 5 min; keep at 4°C indefinitely. The ligation product was purified with beads and then applied on a 1.5% agarose gel. The libraries were quantified with a NanoDrop^@^ instrument, and their concentration was normalized. An equimolar pool was made with all of them. The pool was quantified by qPCR using the KAPA Biosystems kit™ (KAPA Biosystems, USA) to estimate its concentration and load the MiSeq with 20% Phix. The sequencing was performed with paired readings of 2× 250 bp.

### Sequencing analysis

The data were analyzed according to Callahan et al. (2016b) using a set of R packages available through the BioConductor project (Gentleman et al., 2004; Huber et al., 2015). The DADA2 package (Callahan et al., 2016a) was used for trimming and filtering low-quality reads, modeling and correcting amplicon errors, and deduplicating and overlapping the forward and reverse reads. After the removal of chimeric sequences, taxonomies were assigned to each ASV (Amplicon Sequencing Variant) using the DADA2 implementation of the naive Bayesian classifier (Wang et al., 2007), using the Genome Taxonomy Database (GTDB) release 95 as reference (Parks et al., 2017).

Taxonomic classifications generated by DADA2 and their quantifications were imported into the Phyloseq R package (McMurdie and Holmes, 2013). ASVs were grouped at the genera level, and ASVs that were not classified at the family level were filtered out. After these filters, the tables of counts, relative abundance, and Alpha and Beta diversity were obtained. The limma R package (Ritchie et al., 2015) was used to perform the differential abundance analysis. In short, counts were transformed to log2 (counts per million) (Law et al., 2014), which allows the linear models implemented in the limma package to be used to count data. Factors were created for each combination of species + tissue + environment, and the differential taxonomic abundance was tested for each contrast of interest with moderate t-tests (Smyth, 2004). The ordinate function from phyloseq was then used to calculate the Unifrac distance (Hamady et al., 2010), and the graphs of the Principal Coordinate Analysis (PCoA) were generated from these distances (Lozupone and Knight, 2005). Ellipses were computed for the ordination plot with stat_ellipse function from ggplot2 v.3.3.3 (Wickham, 2016), considering a multivariate t-distribution with a 0.95 level. The heatmap analysis was generated using the HTML server http://www.heatmapper.ca/ (Babicki et al., 2016).

Raw sequencing reads were deposited at NCBI Sequence Read Archive under BioProject PRJNA768447. The datasets generated for this study can be found in the NCBI under BioSample accessions SAMN22044346 - SAMN22044369.

## Results

### Characterization of α-diversity

**Figure 3:**
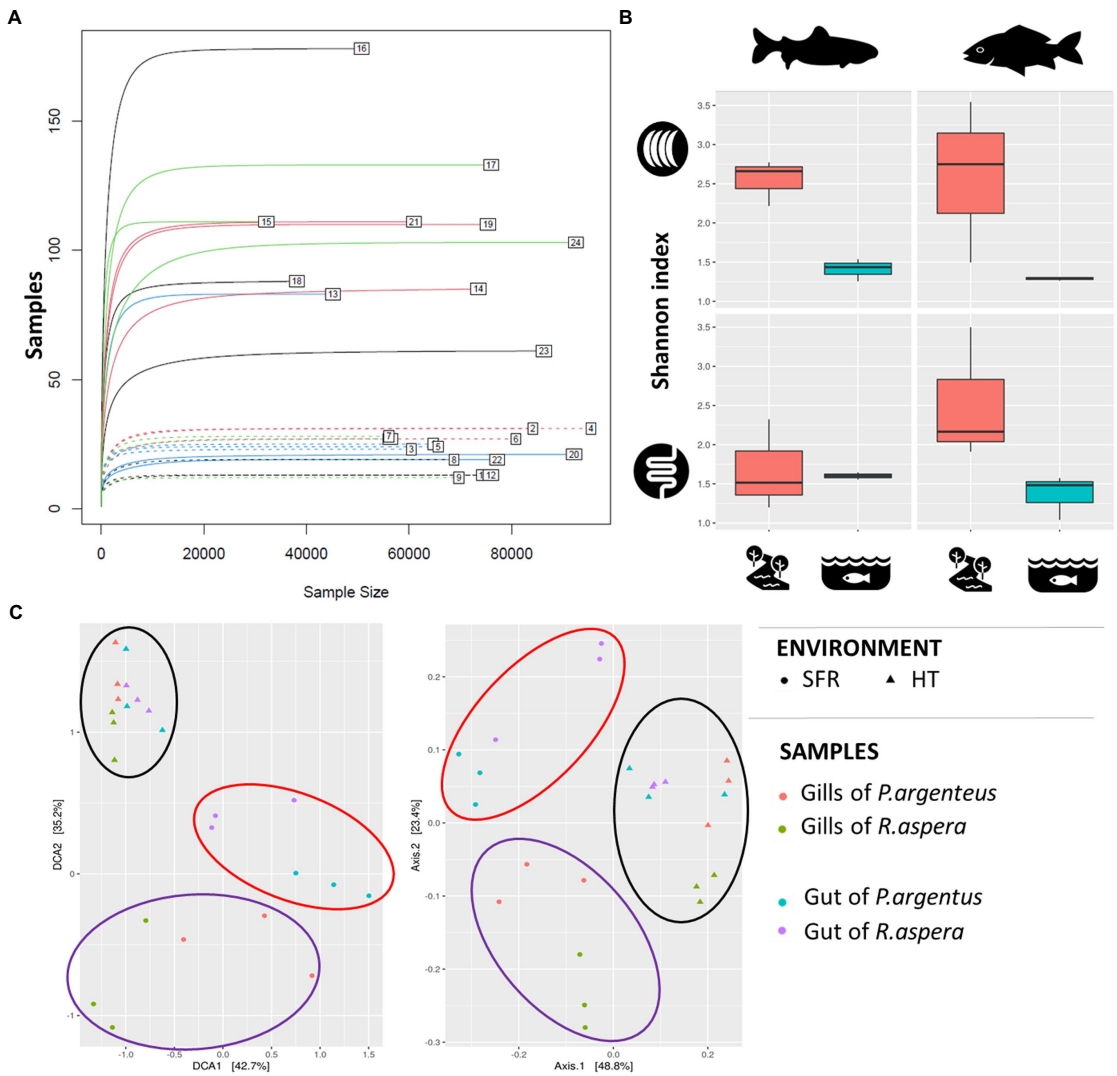
**(A)** Rarefaction curve. The numbers indicate each of the samples described in Table 1. **(B)** Alpha-diversity indices of the microbiota associated with the gills and guts of *R. aspera* and *P. argenteus* from SFR and HT. **(C)** Diversity β is expressed as dispersion, given by the metric of the weighted UniFrac distance to evaluate the similarity between the different species tissues and habitats. Each sample is represented by a triangle (from HT) or a circle (from SFR). The red and blue triangles and circles inherit from the gills and guts of *P. argenteus*, while those from green and purple nuclei inherit from the gills and guts of *R. aspera*.

The microbiomes associated with the gills and guts of *R. aspera* and *P. argenteus* from the SFR and HT were compared (Figure 3A). Different microbial diversity levels were observed between the gills of fish from the same species originating from the SFR and HT (Shannon’s index [*F* = 0.0006] and Simpson’s index [*F* = 0.01974]). Also, microbiomes from the gills and guts showed higher α-diversity in fish from the river than in the hatchery counterparts (Figure 3B and Supplementary Table 2).

### Characterization of β-diversity

Scatter analysis revealed that the clustering pattern of the microbiome was markedly affected by fish origin – SFR or HT (Figure 3C). In addition, there was a sharp distinction between microbial communities associated with each environmental source of fish for each analyzed organ in both species.

### Taxonomic classification

A total of 33 phyla, 61 classes, 133 orders, 220 families, and 295 genera distributed within the gills and guts of *R. aspera* and *P. argenteus* from the SFR and hatchery systems were resolved. The most abundant phyla were Proteobacteria, Fusobacteriota, Bacteroidota, Planctomycetes, Actinobacteria, Cyanobacteria, and Verrumicrobia. Among these, Proteobacteria and Fusobacteriota were predominant in both fish species (Supplementary Table 3 and Supplementary Figure 1), regardless of the environmental source, with the former being more abundant in the SFR (45.11 and 51.3% from *R. aspera* and 74 and 74.75% from *P. argenteus*), respectively in gills and gut and the latter being more abundant in the HT environment (46 and 49% from *R. aspera* and 42 and 68% from *P. argenteus*, respectively in gills and gut. The Bacteroidota phylum was abundant in the gills of *R. aspera* from SFR (16%) and the guts of *P. argenteus* from HT (8.8%). Besides these shared phyla, an exclusive phylum was detected for the SFR: Cyanobacteria, whose abundance varied from 0.87 to 3% in the gills and guts of both species. Other rare phyla were associated with the gills and gut of both species (Armatimonadota, Firmicutes_C, Gemmatimonadota and Spirochaetota), only with the gut of both species (Acidobacteriota, Chloroflexota-A, Dependentiae, and Desulfobacteriota, Myxococcota), only with the gills of *P. argenteus* (Chloroflexota, Elusimicrobiota, and Desulfuromonadota); only the gut of *P. argenteus* (Binatota and Dormibacterota); only the gut of *R. aspera* (Firmicutes_B); associated with the gills of both species and the gut of *P. argenteus* (Deinoccota) (Supplementary Table 3 and Supplementary Figure 1).

At the genera level, the highest diversity was recorded in fish from the SFR (289) when compared with those from the HT (39) (Supplementary Table 3 and Supplementary Figure 2). Furthermore, among the fish from the SFR, genera diversity was higher in both the gut (138) and gills (155) of *P. argenteus* than in the counterparts (54 and 119, respectively) of *R. aspera* (Supplementary Table 3 and Supplementary Figure 2).

### Microbiome characterization of *Rhinelepis aspera* and *Prochilodus argenteus* × environment

**Figure 4:**
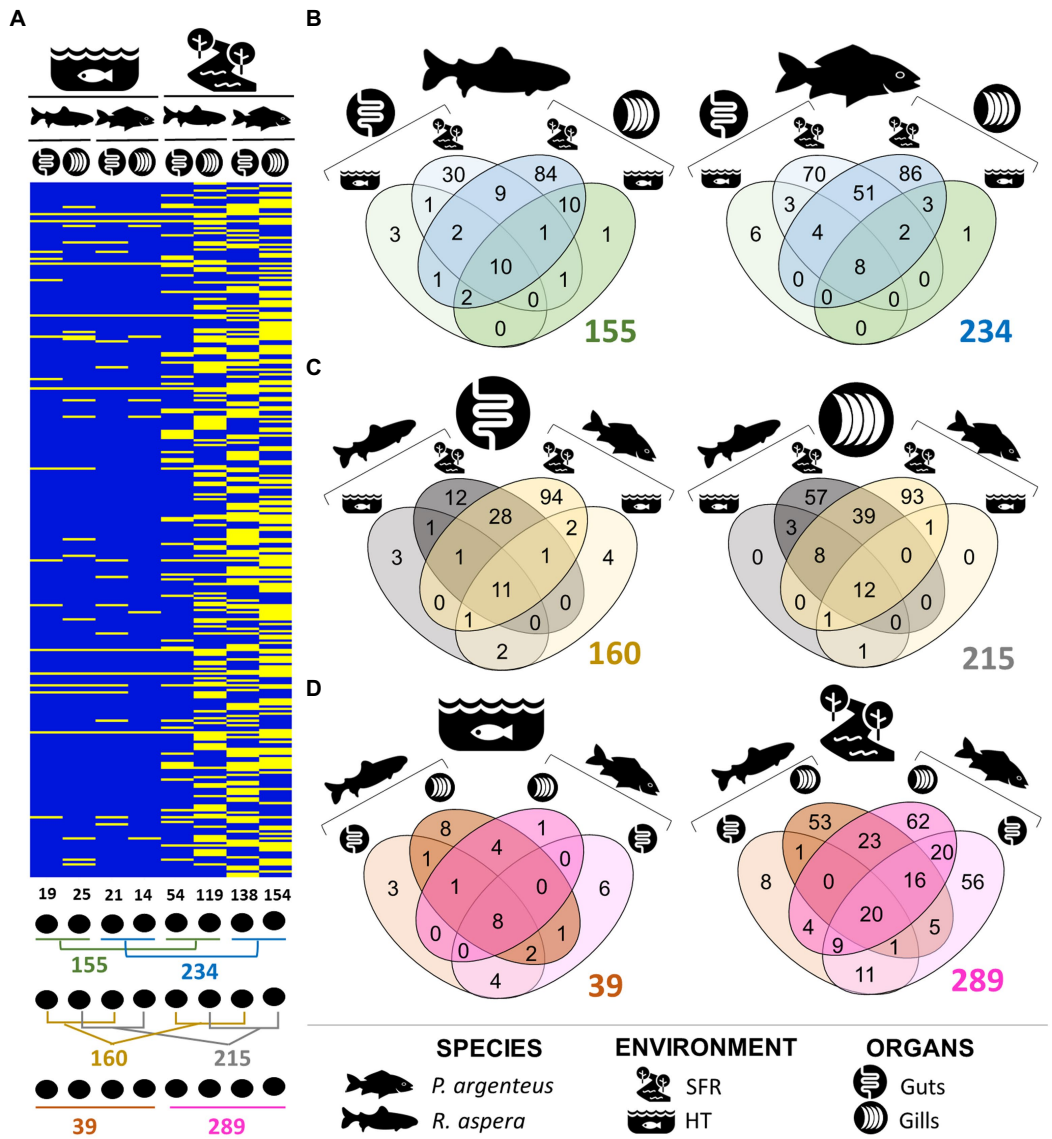
**(A)** Presence and absence of ASVs that attributed taxonomies at the genera level, illustrating the differences in the structure of the gill and intestinal microbiomes of *R. aspera* and *P. argenteus* collected in SFR and HT. Below the heatmap are shown the ways of comparison whose numbers are expressed in Venn diagrams **(B–D)** indicating the number of total, shared, and unique phylotypes in terms of genera level of the microorganisms associated with samples obtained from fish, according to the host species resident in different ecological systems **(B)**, different colonized body niches **(C)** and the host habitats **(D)**.

The number of unique and shared genera of microorganisms among species, environments, and organs can be viewed in Figures 4A–D. Taxonomic details are presented in Table 2. The gills and gut microbiomes of *R. aspera* from both environments showed 155 bacterial taxa for the genera, whereas those of *P. argenteus* had 234 identified taxa (Figures 4A,B). Only 6.45% (10 genera) were shared among organs and environments for the sampled *R. aspera*. In contrast, only 3.41% were common among the 234 identified taxa in the sampled *P. argenteus* (Figures 4A,B).

**Table 2:** Unique communities at the genera level of the gill and gut microbiomes of species recruited from the SFR and HT.

**Environment**	**Species**	**Organs**	**Unique taxons**
SFR	*R. aspera*	Gills	UBA11704, GN02-873, Aalborg-AAW-1, 2-02-FULL-55-14, OLB5, UBA2475, UBA4416, Haliscomenobacter, Taibaiella_B,UBA1931, UBA8137, JJ008, Vibrionimona, Pedobacter, Pseudopedobacter, OLB11, Fluviicola, Flavobacterium_A, Soonwooa, Solirubrum, Flexibacter, Rudanella, Runella, Rhodoluna, Pseudanabaena, Paenibacillus_G, Veillonella, IMCC26134_A, Gemmatimonas), Deinococcus_C, Bacteriovorax, Bdellovibrio_A, Sandarakinorhabdus, Altererythrobacter_B, Porphyrobacter, Cellvibrio, Dechloromonas, Alkanindiges,CAG-495,Thiolinea, Agitococcus, Pseudoxanthomonas_A, 2013Ark19i, Methylophilus, Lautropia, Limnobacter, Pseudoduganella, JOSHI-001, Sphaerotilus, Paucibacter, Hydrogenophaga, Rhodoferax.
		Gut	Blastococcus, Schlegelella, Bartonella,Ga0077555,OLB17, TC1, Planctomicrobium.
	*P.argenteus*	Gills	UBA1568,UBA6899, SZUA-254,Vibrionimonas,Empedobacter, F0040, Porphyromonas, Rufibacter, Spirosoma, 2-02-FULL-39-32, JKG1, Dietzia, Dermacoccus, Curtobacterium, Leucobacter, Pseudonocardia, Nanopelagicus, Planktophila, Obscuribacter, Chroococcidiopsis, Chroogloeocystis, Scytonema_B, Calothrix,UBA7541, Roseburia, Clostridium_J, Anaerococcus, Acetobacterium, Parageobacillus, Piscibacillus, Brevibacillus, Pediococcus,Granulicatella, Abiotrophia, Solobacterium, Pirellula, Sphingopyxis, Caedimonas, Nucleicultrix,Caedibacter, Moraxella_C, Haemophilus_D, Neisseria, Kingella_B, Chromobacterium, UKL13-2, Bordetella, Castellaniella, Ralstonia, Piscinibacter
		Gut	Brevinema, GWE1-39-12, UBA12053, UBA7522, UBA4664, Pseudosphingobacterium, Elizabethkingia, UBA12411,IMCC26256, Marmoricola, Aeromicrobium, Actinomyces, Brevibacterium, Janibacter, Geodermatophilus Planktothricoides, Vulcanococcus, Ruminococcus_E, UBA3792, Clostridium_AN, Neofamilia, Finegoldia, Turicibacter, Lysinibacillus_C, Weissella, Exiguobacterium_A, Exiguobacterium, Centipeda, Dormibacter, Luteitalea, UBA10511, Terrimicrobium, SZUA-320, Oligoflexus, HRBIN30, Anaeromyxobacter, Neorickettsia, Neorhizobium, Rubellimicrobium, Hyphomicrobium, Phenylobacterium, Sphingobium, Sphingomonas_A, Microvirga Z2-YC6860, Alsobacter, Labrys, 2-02-FULL-42-43, Nitrosomonas, Cupriavidus, Sulfuritalea, Lautropia.
HT	*R. aspera*	Gills	None.
		Gut	*Desulfovibrio, Bosea.*
	*P.argenteus*	Gills	None.
		Gut	*UBA5150, Niameybacter.*

### Microbiome characterization of *Rhinelepis aspera* and *Prochilodus argenteus* × organs

The abundance of exclusive genera belonging to the microbiomes associated with different organs in each host species (Figure 4C) indicates the occurrence of interspecific phylogenetic variability that distinguishes the microbiome present in these structures. Furthermore, the distinction between bacterial communities in different organs of *P. argenteus* and *R. aspera* in the SFR was supported by the shared genera proportions of 28.63 and 14.56%, respectively. The same trend of microbial diversity reduction was observed in *P. argenteus* from HT (Figure 4D).

### Microbiota associated only with SFR fishes

The *R. aspera* gill microbiome comprised 21 phyla, 30 classes, 98 families, and 119 genera. Among them, 84 exclusive (54.2%) to SFR (Figure 4B). Proteobacteria (45.41%), Fusobacteriota (11.66%), and Bacteroidota (16%) were the dominant phyla (Supplementary Figure 1). The phylum Fibrobacteriota (<1%) was exclusively associated with the gill microbiota of this species. The phylum Proteobacteria was identified by 68 ASVs that attributed taxonomical classification to 58 genera, with 19 affiliated with Betaproteobacteria, 16 with Gammaproteobacteria, and 13 with Alphaproteobacteria.

The most diverse orders were Burkholderiales, Enterobacterales, and Pseudomonadales. The phylum Bacteroidota was identified by 44 ASVs that classified 33 genera, distributed mainly by the orders Cytophagales, Flavobacteriales, Bacteroidales, and Chitinophagales (Supplementary Figure 1). Interestingly, the phylum Fusobacteriota was identified only by two ASVs, and by an integrated gill microbiome with an average relative abundance of approximately 20%. The phyla Bacteroidota and Proteobacteria comprised 38 exclusive genera to the gill microbiome of *R. aspera,* including *Taibaiella_B*, *Fluviicola*, *Runella*, *Flexibacter*, *Pseudopedobacter*, *Solirubrum*, *Pseudoxanthomonas_A, Porphyrobacter, Cellvibrio, Dechloromonas,* and *Methylophilus*. The most abundant genera were *Aeromonas (18%)*, *Cetobacterium_A* (19.6%), *Flavobacterium* (16.1%), *Shewanella* (5.7%), *Pseudoduganella* (4.34%), *Vogesella* (4.23%), *Acinetobacter* (3%), and *Plesiomona*s (3%) (Supplementary Figure 2).

The gut microbiome of *R. aspera* from the SFR exhibited the lowest observed diversity among other microbiomes in this environment (Figures 4A–C), with 18 phyla, 22 classes, 49 orders, 66 families, and 54 genera, with 30 unique phylotypes (55.55%). The dominant phyla (Supplementary Figure 1) were Proteobacteria (55.44%), identified by 29 ASVs, followed by Fusobacteriota, identified by only three ASVs, representing approximately 28%. Other less abundant phyla included Actinobacteriota (0.72%), Bacteroidota (0.7%), Firmicutes (0.04%), Cyanobacteria (3%), and Planctomycetota (3%). In contrast to the gill microbiome of this species, the phylum Bacteroidota was represented by the families Bacteroidaceae, Weeksellaceae, and Tannerellaceae. The phylum Proteobacteria was diverse, with microorganisms from the class Gammaproteobacteria, from the order Enterobacteriales, and the Aeromonadaceae, Shewanellaceae, and Enterobacteriaceae families, with *Shewanella* (46.17%), *Plesiomonas* (19.48%), and *Aeromonas* (5.74%) standing out in abundance (Supplementary Figure 1). The phyla Firmicutes_A and Firmicutes, represented by the classes Clostridia and Bacilli, respectively, constituted 3.1% of the gut microbiome for this species.

### Tenant microbial communities of *Prochilodus argenteus* from the SFR

The tenant gill microbiome of *P. argenteus* was composed of 23 phyla, 46 classes, 99 orders, 129 families, and 154 genera (Figure 4A), of which 86 (55.84%) were exclusive to this organ (Figure 4B). The dominant phyla were Proteobacteria (74.3%) and Fusobacteriota (14.63%) (Supplementary Figure 1). Some poor abundant phyla were identified by numerically conspicuous sequences, such as Bacteroidota (30 ASVs), Actinobacteria (31 ASVs), and Firmicutes (26 ASVs), which together comprised 58 genera, undoubtedly contributing to increasing microbial diversity but adding less abundant microorganisms to the gill microbiome. Three phyla exclusive to the gill microbiome were observed: Chloroflexota, Desulfuromonadota, and Elusimicrobiota. The most diverse taxa included the genera *Aeromonas* (28.84%), *Cetobacterium*_A (14.63%), *Plesiomonas* (13.33%), *Acinetobacter* (2.94%), *Vogesella* (1.55%), *Polynucleobacter* (1.13%), and other unidentified genera belonging to the Enterobacteriaceae (4%) and Burkhoderiaceae (1.87%) families, in addition to microorganisms exclusive to this organ, *Caedimonas* (6.22%), and *Caedibacter* (2.44%).

The gut microbiome was composed of 26 phyla, 36 classes, 93 orders, 136 families, and 138 genera, with 70 of these genera (50.7%) occurring only for this niche in the host (Figure 4B). The phyla Dormibacteriota and Binatota were present only in the gut microbiome of *P. argenteus*. The dominant phyla were Proteobacteria (47%), Fusobacteriota (25.97%), Planctomycetota (6.2%), Verrumicrobiota (1.7%), and Cyanobacteria (1.46%). Although 31 sequences (ASVs) have been attributed to the phylum Actinobacteria, with 22 genera, this taxon integrated the microbiome with low abundance (<1%). The order Enterobacterales and Fusobacteriales contained the most abundant genera, *Aeromonas* (7.55%), *Plesiomonas* (23.24%), and *Cetobacterium*_A (25.9%), respectively. From the phylum Spirochaetes, the family Brevinemataceae was recorded only in the gut microbiome of *P. argenteus*. The phylum Dependentiae was observed as part of the intestinal microbiome of both *R. aspera* and *P. argenteus,* collected from the SFR.

### Microbiota associated only with HT fishes

The gill microbiota of *R. aspera* reared in HT consisted of 8 phyla, 24 families, 15 orders, 9 classes, and 25 genera. The dominant phyla (Supplementary Figure 1) were Fusobacteriota (49%), Proteobacteria (24.6%), and Bacteroidota (0.76%). Less abundant phyla (<1%) included Actinobacteria, Bdellovibrionata, Firmicutes, Patescibacteria, and Verrumicrobiota. The phylum Firmicutes was represented by a single family, Erysipelotrichaceae, and by a single genus, *Anaerorhabdus*. The phylum Campylobacterota showed only the genera *Aliarcobacter*, constituting an exclusive taxon to the gill microbiome of *R. aspera* and *P. argenteus*. We found 9 phyla, 10 classes, 16 orders, 21 families, and 19 genera in the gut microbiome. In addition, there were two unique and less abundant genera (<1%), *Bosea* and *Desulfovibrio* (Table 2). The samples isolated from both niches were dominated by Fusobacteriaceae and Enterobacteriaceae families. At the genera level, similarly to the fish obtained from the SFR, the gills and gut microbiome were dominated by *Cetobacterium*_ A (49 and 46%), *Plesiomonas* (15.7 and 27.9%), and *Aeromonas* (24 and 5%), *Edwardsiella* (1.12 and 1.59%) and *Bacteroides* (0.82 and 1.9%), respectively (Supplementary Figure 2). The Anderseniellaceae family was observed at a higher relative abundance (7.5%), integrating the gut microbiome of *R. aspera*.

The gill microbiota consisted of 7 phyla, 7 classes, 11 orders, 15 families, and 14 genera (Supplementary Figure 1). The structure of the gill and gut microbiomes was strongly modulated by the environmental conditions imposed by HT (Figure 4A). However, the gill and gut microbial communities did not differ significantly in their taxonomic ASV identities.

**Figure 5:**
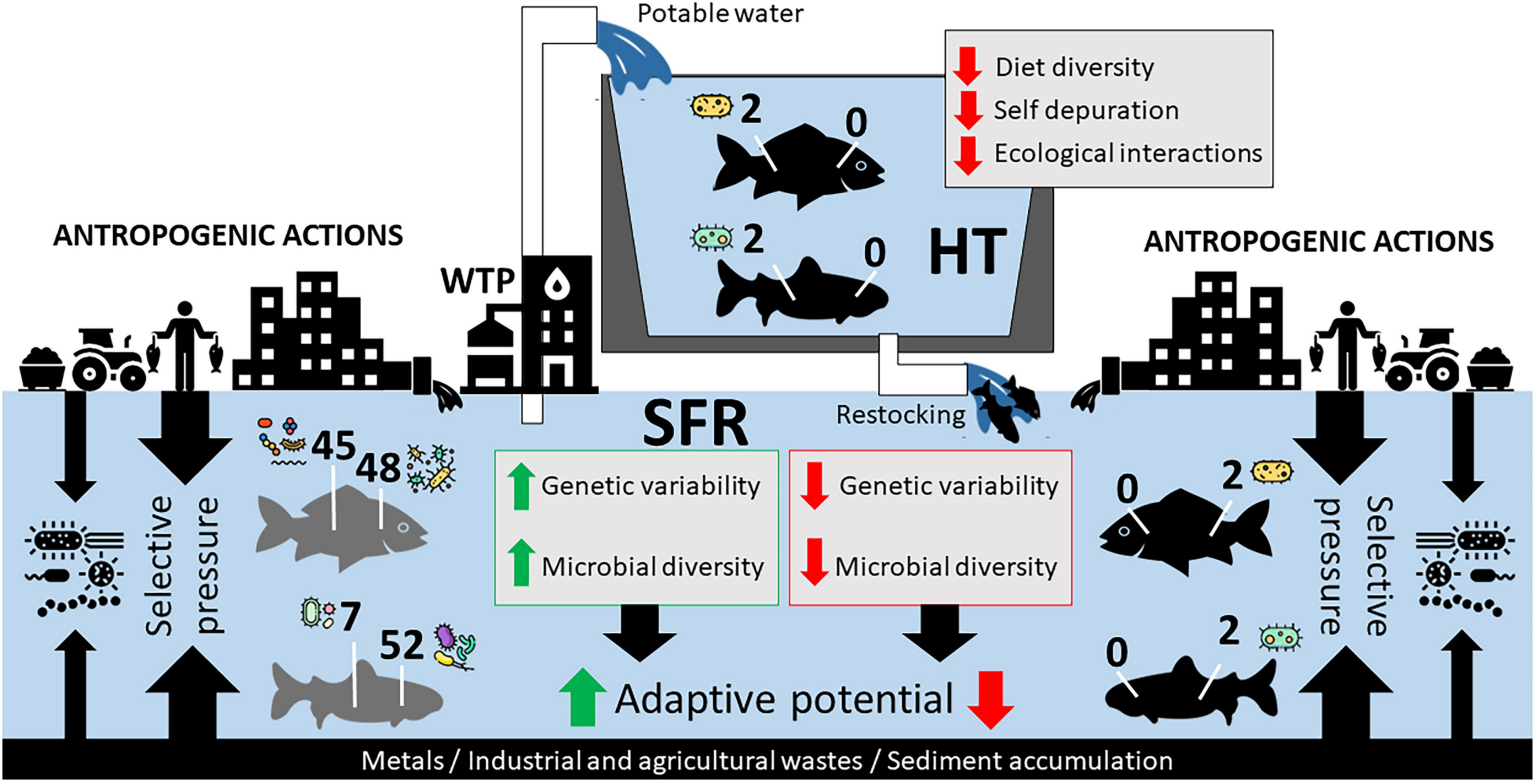
Hypothetical model of the adaptive relationship of species in SFR, in HT, and after being returned to SFR after restocking. Impacts of anthropogenic actions in SFR and zootechnical management in HT on the constitution of *R. aspera* and *P. argenteus* microbiomes should contribute to microbiota structure. The reduction of genetic diversity and the gill and gut microbiota can massively interfere with the adaptation of fish after restocking.

The intestinal microbiota was composed of 7 phyla, 10 classes, 17 orders, 24 families, and 21 genera, and among these, 6 exclusive genera were identified (Figure 4B). The dominant and most diverse phyla on the gill microbiota and the gut microbiota of *P. argenteus* were Proteobacteria (51 and 12.79%) and Fusobacteriota (44 and 60.82%, respectively). In the gills, the genera *Aeromonas* (16.9%), *Cetobacterium*_A (42%), and *Plesiomonas* (33.40%) dominated the microbial assemblage (Supplementary Figure 2). The gut presented a reduced relative abundance of the genera *Aeromonas* (3.66%) and *Plesiomonas* (12.79%), as well as an increase in *Bacteroides* (8.37%) and *Edwardsiella* (3.96%) (Figure 5).

### Unique microbiota associated with organs

Of the 295 bacterial genera identified among all the different samples, 215 were reported to be associated with gills (Figure 4C), with 12 genera (5.58%) shared among all samples originating from the gills (SFR and HT). Of these, 214 were associated with the gill microbiomes of the fish recruited from the SFR (119 in *R. aspera* and 154 in *P. argenteus*), both of which shared 27.5% in common. The interspecific difference in the gill microbial community composition was evidenced by the number of exclusive genera in *R. aspera* (54) and *P. argenteus* (93). The dominant genera in both species included *Cetobacterium*_A, *Aeromonas*, and *Plesiomonas* (Supplementary Figure 2).

Although present in both species, the genera *Flavobacterium*, *Shewanella*, *Vogesella*, and *Pseudoduganella* were more abundant in the gills of *R. aspera* from the SFR (Supplementary Figure 2). Some of the exclusive taxa from the gill microbiome (Table 2) were mainly classified within the Bacteroidetes phylum (*Fluviicola*, *Flavobacterium*, *Soonwooa*, *Solirubrum*, *Haliscomenobacter, Taibaiella*, *Pedobacter*, *Sphingobacterium*, and *Flexibacter*), Betaproteobacteria (*Rhodoferax*, *Hydrogenophaga*, *Paucibacter*, *Sphaerotilus*, *Limnobacter)* and Gammaproteobacteria (*Alkanindiges, Cellvibrio, Pseudoxanthomonas, Thiolinea*).

Some of the unique microbial taxa associated with the gills of *P. argenteus* included *Caedimonas*, *Caedibacter*, *Vibrionimonas*, *Porphyromonas*, *Nanopelagicus*, *Planktophila*, *Scytonema, Dermacoccus, Dietzia*, *Granulicatella*, *Rufibacter*, *Leucobacter*, *Curtobacterium*, *Moraxella_C*, *Acetobacterium*, *Haemophilus*, *Chromobacterium*).

Reduced microbial diversity was associated with gills from both species recruited from the HT, with more remarkable interspecific similarity (53.84% of shared genera) of the identified microorganisms. The proportion of commonly shared genera in comparing the same species (SFR vs. HT) was 9% for *P. argenteus* and 19% for *R. aspera* (Figure 4C). The gill microbiome was dominated by the Fusobacteriaceae, Enterobacteriaceae, Aeromonadaceae, and Bacteroidaceae families.

### Microbiota communities associated with medium gut

The patterns of interspecific variations became conspicuous when comparing bacterial communities colonizing the medium gut of *P. argenteus* and *R. aspera* from SFR, which amounted to the lowest microbial diversity of the characterized microbiomes from this environment. We registered 151 genera, with 7.28% of this total shared among the two host fish species (Figure 4C).

The microbial community in the guts of *P. argenteus* comprised 138 genera, of which 94 (67.4%) were exclusive to that host (Figure 4C). These results clearly distinguish the intestinal microbiota of the evaluated fish species. The intestinal microbiota in both fish, recruited from SFR and HT, was predominantly composed of the microorganisms *Cetobacterium*_A, *Aeromonas*, and *Plesiomonas*. Differences were noted in the higher abundance of the *Shewanella* genera (46.17%), integrating the intestinal microbiota of *R. aspera* from the SFR. The *Edwardsiella* genera was observed over the gills and guts of both species, varying in abundance from 0.45 to 3.96%, with more significant proportions found in the samples of hosts reared in HT. Among the unique genera associated with the enteric microbiota of *P. argenteus* (Table 2), we observed microorganisms affiliated with the classes Alphaproteobacteria (*Rubellimicrobium, Hyphomicrobium, Phenylobacterium, Sphingomonas_A, Microvirga*) and Betaproteobacteria (*Nitrosomonas, Cupriavidus, Sulfuritalea, Lautropia*).

The HT environment also negatively affected the gut microbiome composition in both species, with only 19 and 21 genera colonizing the guts of *R. aspera* and *P. argenteus*, respectively. However, the relatively small number of exclusive taxa (Figure 4C) in *R. aspera* (3) and *P. argenteus* (4) indicates that there is little interspecific variability in the microbial diversity of the guts of both host species.

### Phylogenetically distinct host species share a central microbiome in different ecological systems

The central microbiota comprised the proportion of the microbial community shared among all samples. The gill and gut microbiomes of both species share many broader taxonomic groups. At the genera level, the gill and gut microbiomes shared between *R. aspera* and *P. argenteus* were remarkably low, accounting for only 3.4 to 6.92% of the total when compared between fish species and environments. Interestingly, most diverse genera, like *Aeromonas* (3.6 to 28.84%), *Cetobacterium* (19 to 60.82%), and *Plesiomonas* (3 to 33.40%), were shared between organs and species of the SFR and the HT (Supplementary Figure 2). Other taxa shared at the genera level at least 75% of the total biological samples included *Bacteroides*, *Akkermansia*, *Edwardsiella*, *Pseudomonas*, and *Shewanella,* among other non-identified associated with Burkholderiaceae, Enterobacteriaceae e Fusobacteriaceae families.

## Discussion

SFR is one of the most important Brazilian rivers. It contributes to the national energy matrix, favoring tourism and agriculture in its surroundings and providing fish to those who depend on it (Sun et al., 2016; Castro and Pereira, 2019). However, throughout their extension, these activities have contributed to the decline of the ichthyofauna, especially related to endemic and native species to this watershed (Holanda et al., 2009; Barbosa et al., 2017; Figueiredo, 2018).

This worrying scenario, which repeats in other basins around the world, puts aquaculture as a positive contributor to increasing the supply of fish as a source of animal protein (FAO, 2016). That minimizes the pressure on population stocks at the same time that it can support stochastic re-population strategies to preserve biodiversity (Klinger and Naylor, 2012).

However, studies indicate that the release of fish cultivated in re-population programs can pose risks of disease transmission to native populations due to the decrease in genetic variability and the possible introduction of exotic alleles that can compromise wild populations (Araki and Schmid, 2010; Cheng et al., 2015; Pavlova et al., 2017; Savary et al., 2017). Additionally, very little is known about the microbiomes associated with wildlife. Therefore, the first characterization of the structure of not yet known microbiomes associated with fish species may reveal an as-yet undescribed and particular microbiota (Levin et al., 2021). Furthermore, there is limited research on the relationship between species confinement and the modulation of microbiota associated with important tissues such as skin, gills, and guts.

In an attempt to reduce this knowledge gap and effectively contribute to studies involving fish species of ecological and commercial interest, this work characterized and compared for the first time the intestinal and gill microbiota of the species *P. argenteus* and *R. aspera* from the SFR and kept in an HT, both endemic to SFR natives, by high-throughput DNA sequencing. This culture-free method allows a broader view of microbial diversity associated with organisms, organs, and environments.

The present research findings indicate that fish-rearing conditions significantly altered the structure of the gut and gill microbiome of *R. aspera* and *P. argenteus* from the HT, suggesting that it was strongly modulated by the environmental conditions imposed by the host habitat. The microbiomes of the confined fish showed an 87% reduction in the diversity of taxa compared with the same species collected from the river. Studies show that the composition of microbiomes in teleost fish can differ under the influence of several factors such as phylogenetic position, trophic level and diet composition, the microhabitat used by hosts, its environment, and the stocking and rearing densities in hatchery environments (Sullam et al., 2012; Ursell et al., 2012; Boutin et al., 2013; Sylvain et al., 2016; Uren Webster et al., 2018, 2020; Tarnecki et al., 2019). In this context, dysbiosis caused by the drastic decrease in microbial diversity due to environmental variables and changes in diet can affect the nutritional and immunological status of fish, making them more vulnerable and susceptible to opportunistic pathogens (Boutin et al., 2013; Pękala-Safińska, 2018) and compromising aquaculture and food security.

The structure of microbiomes from fish confined in HT showed greater uniformity, with the predominance of the phyla Fusobacteriota and Proteobacteria, which affiliated with the majority (> 95%) of the identified taxonomic signatures. These results are consistent with other findings in other fish species evaluated in a confinement system, such as the cultivation of *Panaque* sp., *Lates calcarifer*, *Oreochromis niloticus*, and *Arapaimas gigas* (Di Maiuta et al., 2013; Zhai et al., 2017; Ramírez et al., 2018; Xia et al., 2020). In contrast, the wild fish microbiomes (SFR) showed greater richness and diversity, mainly composed of Proteobacteria of the classes Alphaproteobacteria, Betaproteobacteria, and Gammaproteobacteria, followed by Fusobacteriota, Bacteroidota, Planctomycetota, and Cyanobacteria. The environmental heterogeneity expected from the river (more so given its diffuse anthropic impacts) should impact fish-associated microbiomes (Krotman et al., 2020). Being illiophagous, both fish grate the substrate and feed over fresh and decomposing algae and also have an intimate relationship with a more anoxic layer of the benthos, where a whole community of specialized microorganisms would be expected.

It is increasingly evident that microbiomes associated with wild animals provide these hosts with metabolic adaptations for survival in environments contaminated by recalcitrant pollutants and the consumption of toxic foods or those deteriorated by pathogens (Tarnecki et al., 2019; Levin et al., 2021). At the same time, some taxa can be characterized as possible biomarkers of environmental pollution (Caruso, 2016; Sylvain et al., 2016; Ventorino et al., 2018; Tarnecki et al., 2019; Nolorbe-Payahua et al., 2020). *Cetobacterium*, *Aeromonas*, and *Plesiomonas* also enriched SFR and HT fish microbiomes. The predominance of *Cetobacterium* was notably observed as associated with the gills and gut of *P. argenteus* and *R. aspera* collected from HT and has also been reported for other commonly cultivated fish species such as *Cyprinus carpio* (van Kessel et al., 2011), *Panaque nigrolineatus* (Di Maiuta et al., 2013), *Ictalurus punctatus, Micropterus salmoides*, *Lepomis macrochirus* (Larsen et al., 2014), *Arapaimas gigas* (Ramírez et al., 2018) and *Oreochromis niloticus* (Yu et al., 2019). The genera *Aeromonas* and *Plesiomonas* were prevalent in all samples, with a higher relative abundance of *Aeromonas* in the gills of *R. aspera* (24%) and *P. argenteus* (28.43%) from the HR and SFR, respectively, contrasting with the reduced abundance associated with intestinal samples (between 3.66 and 7.55%) in both hosts recruited from the SFR and HT. The genera *Aeromonas* is highly pathogenic, comprising 36 genera that establish symbiotic and pathogenic interactions with their hosts (Fernández-Bravo and Figueras, 2020). They carry multiple virulence factors (Nam and Joh, 2007) and are resistant to antimicrobials (Cízek et al., 2010; Nguyen et al., 2014) and heavy metals (Akinbowale et al., 2007). *Plesiomonas* is commonly found in the aquatic environment and associated with fish, being recognized as potential enteropathogens (Pękala-Safińska, 2018). *Plesiomonas* integrated the SFR and HT fish microbiota in relative abundance from 3% gill of *R. aspera* from SFR to 33.4%. These results corroborate those reported for *Plesiomonas* commonly found in fish microbiota (Duarte et al., 2014; Mohammed and Arias, 2014), with a higher prevalence rate in fish spawn in aquaculture.

The most diverse taxa of the microbiomes characterized in this study for *R. aspera* and *P. argenteus* were *Aeromonas*, *Flavobacterium*, *Acinetobacter*, *Plesiomonas*, and *Shewanella*, which frequently colonize the mucous surfaces of fish and are associated with severe pathologies in these hosts (Wu et al., 2012; Latha and Mohan, 2013; Liu et al., 2016; Tarnecki et al., 2016; Pękala-Safińska, 2018; Pratte et al., 2018). In this context, Sylvain et al. (2016) considered *Flavobacterium* a natural biomarker of environmental stress, susceptible to acidic pH, in the context of its abundance decrease, in the skin mucus and feces microbiome of *Colossoma macropomum* while exposed to ever-increasing acid concentrations. In contrast, Yu et al. (2019) demonstrated that exposure of Nile tilapia to aluminum significantly increased the abundance of *Flavobacterium* and decreased *Plesiomonas*. *Acinetobacter* was detected in greater quantities, integrating both wild species’ gill and gut microbiomes. They are reported as emerging pathogens, often associated with polluted environments, carrying antimicrobial resistance genes, which have also been reported as alkane degraders (Manchanda et al., 2010; Costa et al., 2015) and have been isolated from *Oncorhynchus mykiss* and *Cyprinus carpio*, causing severe damage to organisms (Pękala-Safińska, 2018). The genera *Shewanella* was observed in 46% of the guts of wild *R. aspera*, which was different from that reported by Pratte et al. (2018), where it was among the most diverse OTUs in gill microbiomes of 15 species of teleost fish recruited from coral reefs. Bacterial strains of the genera *Shewanella* have been intensively researched as candidates for biotechnological applications, especially in the bioremediation of hydrocarbons and metallic pollutants (Lemaire et al., 2020), and also have been reported to cause infections in popular freshwater-reared species *Cyprinus carpio* and *Oncorhynchus mykiss* (Pękala et al., 2015). The genera *Edwardsiella* is frequently reported to be associated with fish microbiota and includes pathogens such as *E. tarda* and *E. ictaluri* (Pękala-Safińska, 2018); and is also related to the massive mortality of *Myleus micans* (pacu), an important fisheries resource in the SFR, that occurred in the turn of 2005/2006 (Lima et al., 2008). The present study shows that the microbiota of these fish confined in this hatchery system had an increased abundance of *Edwardsiella* spp. (0.84% a 3.96%); therefore, it is a potential genus to risk the aquaculture production of the investigated species.

The phylum Cyanobacteria was observed only in samples originating from wild fish, aggregating 11 genera, among which *Planktothrix* and *Pseudanabaena* are frequently related to flowering and production of cyanotoxins (Su et al., 2015) in eutrophic environments with high concentrations of nitrogen and phosphorus (Paerl, 2018). These results corroborate those presented by the *Instituto de Gestão da Águas de Minas Gerais* (IGAM, 2018), indicating frequent events of bacterial blooms in the middle SFR. Bacteroidetes of the order Chitinophagales and Cytophagales were prevalent and more abundant in the gill microbiota of *R. aspera* in the SFR, and together, they affiliated with nine unique genera. The Chitinophagales family was associated with environments contaminated by heavy metals and impacted by agricultural activities (Costa et al., 2015; Júnior et al., 2021), which are also configured as common scenarios for the SFR.

Some taxonomic signatures were prevalent in the different body niches of both species, recruited from their natural habitat and captivity under the microbiomes characterized in this study. Therefore, we corroborate the hypothesis that phylogenetically diverse species may share basal or nuclear microbiomes (Roeselers et al., 2011; Lowrey et al., 2015; Hennersdorf et al., 2016; Liu et al., 2016). Interestingly, these shared taxonomic signatures correspond to 2.65% of the total chains from the *R. aspera* and *P. argenteus* microbiome, the most abundant taxa found in the samples: *Cetobacterium*, *Plesiomonas*, and *Aeromona*s. Finally, in this study, no significant differences were found in the diversity index α (Shannon and Simpson) between the compared microbiomes, considering species and organs of fish collected in the SFR. This result suggests that the sympatric occurrence of *R. aspera* and *P. argenteus* is associated with the iliophagous ecological convergence and has contributed to the microbial similarity observed in phylogenetically distinct fish species. Previous studies on the characterization of bird, mammal, and fish microbiomes have described the overlap of the ecological niche with increased interspecific microbial convergence (van Veelen et al., 2017; Baldo et al., 2019; Song et al., 2020).

However, a particular distinction between the microbiomes of different tissues of wild fish can be supported by the expressive number of unique taxa found only in the hosts’ gills and guts and by the percentage of shared taxa. In *P. argenteus*, only 28.6% of the taxa at the genera level were shared between gills and guts. This result corroborates the findings of Pratte et al. (2018), which characterized microbiomes of different populations of fish recruited from coral reefs, reporting between 20 to 25% of taxa shared between the gill and intestinal microbiota of individuals of the same species. The intestinal microbiota of *R. aspera* obtained from the SFR harbored a lower richness of bacterial consortia considering this environment compared to *P. argenteus*, also showing a high intraspecific variation between the gill and gut microbiota by sharing only 14.56% of genera. A similar result was reported for other Siluriformes related to the enteric microbiota, mucus, and gut content of *P. nigrolineatus*, *Hypostomus auroguttatus*, and *Pelteobagrus fulvidraco* (Wu et al., 2010, 2012; Duarte et al., 2014). Therefore, the observed variations may be directly related to the phylogeny and diet of the hosts, as well as the role that these tissues play in fish (Ngugi et al., 2017; Pratte et al., 2018; Levin et al., 2021).

From the perspective of fish farming for the repopulation of endangered species, our study brings a new discussion to the debate. The confinement of fish of the species *R. aspera* and *P. argenteus* resulted in a considerable discrepancy between the microbiota from wild and domesticated animals, with a massive reduction of bacterial diversity both in the gills and in the gut of HT fish. This effect probably reflects the contrasting selective pressures of the farming system and the natural habitat and points to vulnerabilities to the aquaculture of these species. Microbiomes as small as those observed in this study in confined fish can negatively impact the hosts’ health, resulting in economic losses and a possible reduction in the ability of cultured fish to be reintroduced into nature to rebuild wild populations (Figure 5). This effect, associated with a decrease in the genetic variability of individuals in confinement, can lead to high production costs with low adaptive capacity in the wild, thus opening new investigative windows in proposals for reintroducing native and endemic species.

One of the biggest challenges to the growth of industrial aquaculture is disease control (Zheng et al., 2020; Karvonen et al., 2021). In this context, the commensal microbiota is important in the innate and adaptive immunity of animals (Vadstein et al., 2018; Sehnal et al., 2021; Huang et al., 2022), whose change in composition can promote or mitigate disease states in hosts (Sehnal et al., 2021). Thus, the reduction in gut and gill microbiome diversity reported in fish farmed in closed systems may have epidemiological relevance and should not be overlooked (Pękala-Safińska, 2018; Tarnecki et al., 2019; Amillano-Cisneros et al., 2022). This perspective is supported by similar studies in *Cyprinus carpio* (Ruzauskas et al., 2021), *Chirostoma estor* (Amillano-Cisneros et al., 2022), *Tor tambroides* (Tan et al., 2019), *Salmo salar* (Uren Webster et al., 2018; Lavoie et al., 2021), *Centropomus undecimalis* (Tarnecki et al., 2019), and *Paralichthys adspersus* (Salas-Leiva et al., 2017).

Recent research, conducted mainly in mammals, has demonstrated the importance of microbiota-host interactions, including the development and regulation of the host’s immune response in the fight against infections and diseases. Reports in the literature relate the loss of microbial diversity in fish exposed to stress or disease (Legrand et al., 2020). In this context, exposure of *D. rerio* to the pathogen *Aeromonas hydrophila* decreased the abundance of beneficial microbial species and increased the abundance of harmful microorganisms and reactive oxygen species (Yang et al., 2017). Inflammatory disorders such as intestinal enteritis have been affecting the rearing of several fish species and were positively correlated with dietary supplements enriched with soybean meal and changes in the structure and diversity of the host microbiota (Urán et al., 2008; Legrand et al., 2020). Intestinal enteritis-induced Mycoplasmataceae enrichment and drastic loss of skin diversity were found in sick yellow-tailed kingfish (Legrand et al., 2020). Such changes can lead to functional dysbiosis in the host and loss of resistance to the invasion of opportunistic pathogens (De Schryver and Vadstein, 2014), demonstrating that the disruption of the host’s microbial flora homeostasis makes it more susceptible to diseases. Bacterial phyla associated with the gut microbiota that metabolize steroid hormones, including progesterone, estradiol, and testosterone, have also been reported in several animal models (Hussain et al., 2021), thus evidencing the involvement of the intestinal microbiota in reproduction. Lavoie et al., 2021 pointed out the relevance of monitoring bacterial communities of fish kept in captivity for repopulation programs, as they observed that the microbiota of confined fish remained different from that of wild fish, even after 2 years of translocation, with resistance to colonization by bacterioplankton bacteria in the natural habitat. Even though this study resolves fish-associated microbial diversity at the genera level, it is reasonable to assume many of the ASVs listed here represent new species. Thus, this line of inquiry would benefit from the whole assembly metagenome in the future to reveal this hidden biodiversity (Levin et al., 2021).

Our study revealed that wild species harbor highly diverse microbial communities. This repertoire of information will contribute to the understanding of microbiota-host bioecological interactions. Additional studies are needed to investigate the functional profile of wild microbiota on fish health and assess the impacts of reduced microbial diversity on farmed fish. Such information will constitute a reference for future studies, either as an attribute of comparing information to other systems and biological models or for implementing improvements in fish production for consumption or repopulation of natural environments.

In conclusion, the stress conditions the fish from HT are subjected to negatively modify the composition of the microbiomes of the host fish from both species, with a significant loss of diversity compared to the microbiomes of their wild counterparts. The set of bacterial communities associated with the body niches of both fish species reared in tanks points to a picture of dysbiosis with a possible decrease in the immune response of host fish and low resistance to colonization by pathogens, which can cause significant losses to aquaculture. We also identified taxa where the investigated species can establish important symbiotic interactions such as nutrition, detoxification, and protection. However, genera frequently reported as opportunistic pathogens were also associated with the investigated tissues, causing public health risks because they are conveyed in water and various foods. This study is the first to characterize the structure and composition of microbial diversity in *R. aspera* and *P. argenteus via* high-throughput sequencing of the 16S rRNA gene. These results can be used as a departure point to evaluate the putative effects of stocking impacted environments with fish spawn in hatcheries in the context of conservation, fisheries, and fish production in the SFR.

## Data availability statement

The datasets generated for this study can be found in the NCBI under BioSample accessions SAMN22044346 - SAMN22044369 - https://www.ncbi.nlm.nih.gov/biosample/22044369 to https://www.ncbi.nlm.nih.gov/biosample/22044369.

## Ethics statement

The animal study was reviewed and approved by this work has an environmental licensing nos. 45371-2 and 59845-1 issued by the Ministry of the Environment–MMA, Chico Mendes Institute for Biodiversity Conservation–ICMBio, through the Biodiversity Authorization and Information System-SISBIO and was approved by the Ethics Committee at Use of Animals CEUA / UFOP by protocol no. 2017/54 and enrolled in SISGEN A68C86F.

## Author contributions

MD, CC, LB, and CL collected fish from the São Francisco River and the polyculture system in a suspended tank. MD, CL, LB, CG, and LM conceived and designed all the experiments. PT, HM, LM, CG, MD, and CL analyzed the biological data. NGS genomic solutions, Microbiology Laboratory of the Federal Institute of Education, Science, and Technology of Northern Minas Gerais contributed reagents, materials, and analysis tools. PT, HM, LM, and MD prepared the figures and tables. MD, CL, CG, LM, PT, HM, AS, and LR wrote, revised, and approved the final version of the paper. All authors contributed to the article and approved the submitted version.

## Funding

This study was financed in part by the Coordenação de Aperfeiçoamento de Pessoal de Nível Superior – Brazil (CAPES) – Finance Code 001 - (the BIGA Project, CFP 51/2013, process 3385/2013 and DINTER Project Biological Sciences 223616/2014), National Council of Technological and Scientific Development (CNPq Process 481226/2013-3), and by the Fundação de Amparo à Pesquisa do Estado de Minas Gerais (FAPEMIG process APQ-02357-17). LM has a research fellowship from CNPq. CG and LM have UFOP grants.

## Conflict of interest

The authors declare that the research was conducted in the absence of any commercial or financial relationships that could be construed as a potential conflict of interest.

## Publisher’s note

All claims expressed in this article are solely those of the authors and do not necessarily represent those of their affiliated organizations, or those of the publisher, the editors and the reviewers. Any product that may be evaluated in this article, or claim that may be made by its manufacturer, is not guaranteed or endorsed by the publisher.
