# Supplementary material for: Hatchery tanks induce intense reduction in microbiota diversity associated with gills and guts of two endemic species of the São Francisco River
Source: Front Microbiol. 2022 Dec 2;13:966436. doi: 10.3389/fmicb.2022.966436 (PMC9755704; doi:10.3389/fmicb.2022.966436)
Supplement: Supplementary file 1 [file Data_Sheet_1.doc]

Supplementary Material


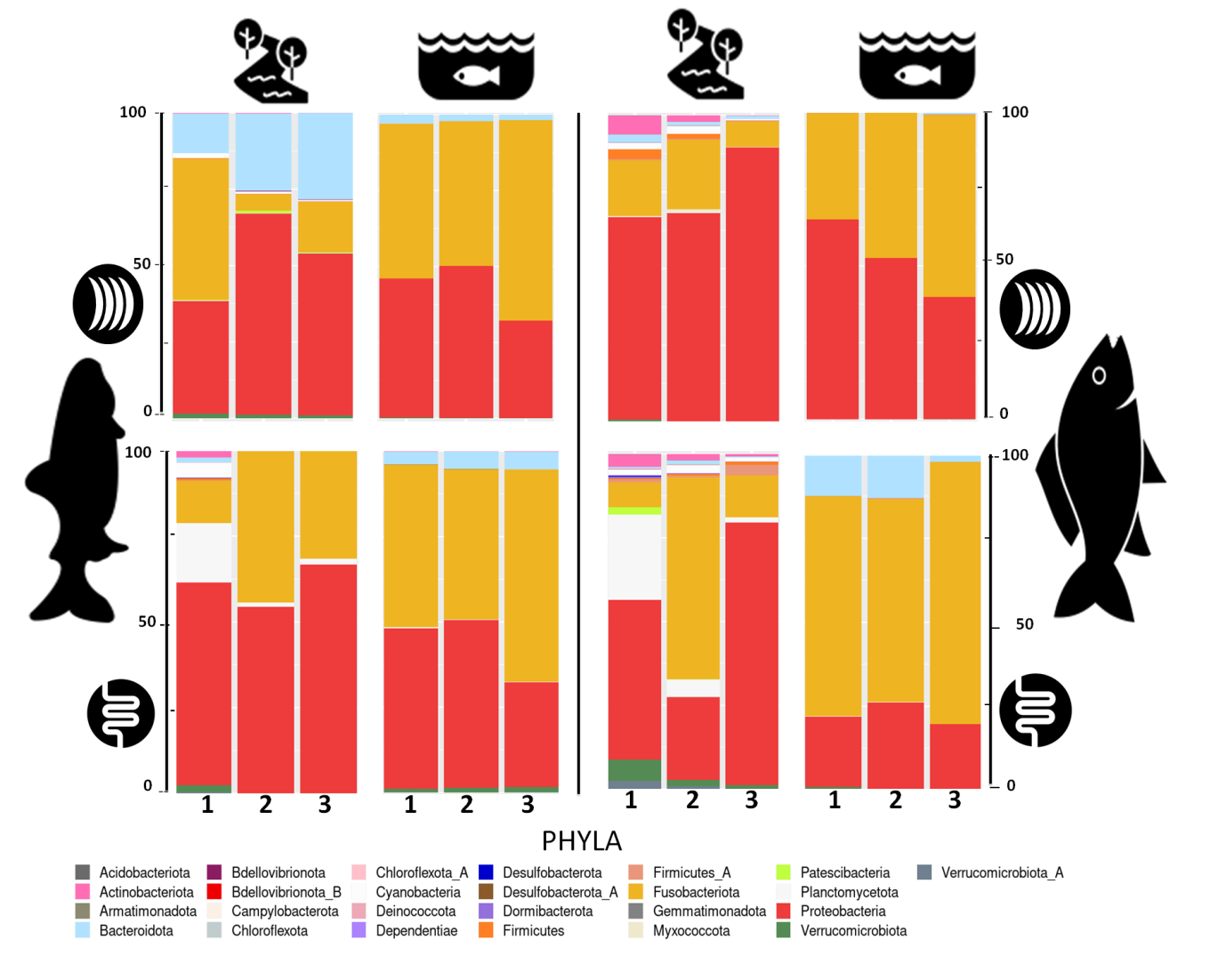


**Supplementary Figure 1.** Microbial diversity at phylum level found associated with samples obtained from the gills and intestines of iliophage / dedritivorous fish from the ichthyofauna of the São Francisco River and an intensive polyculture system in a suspended tank.


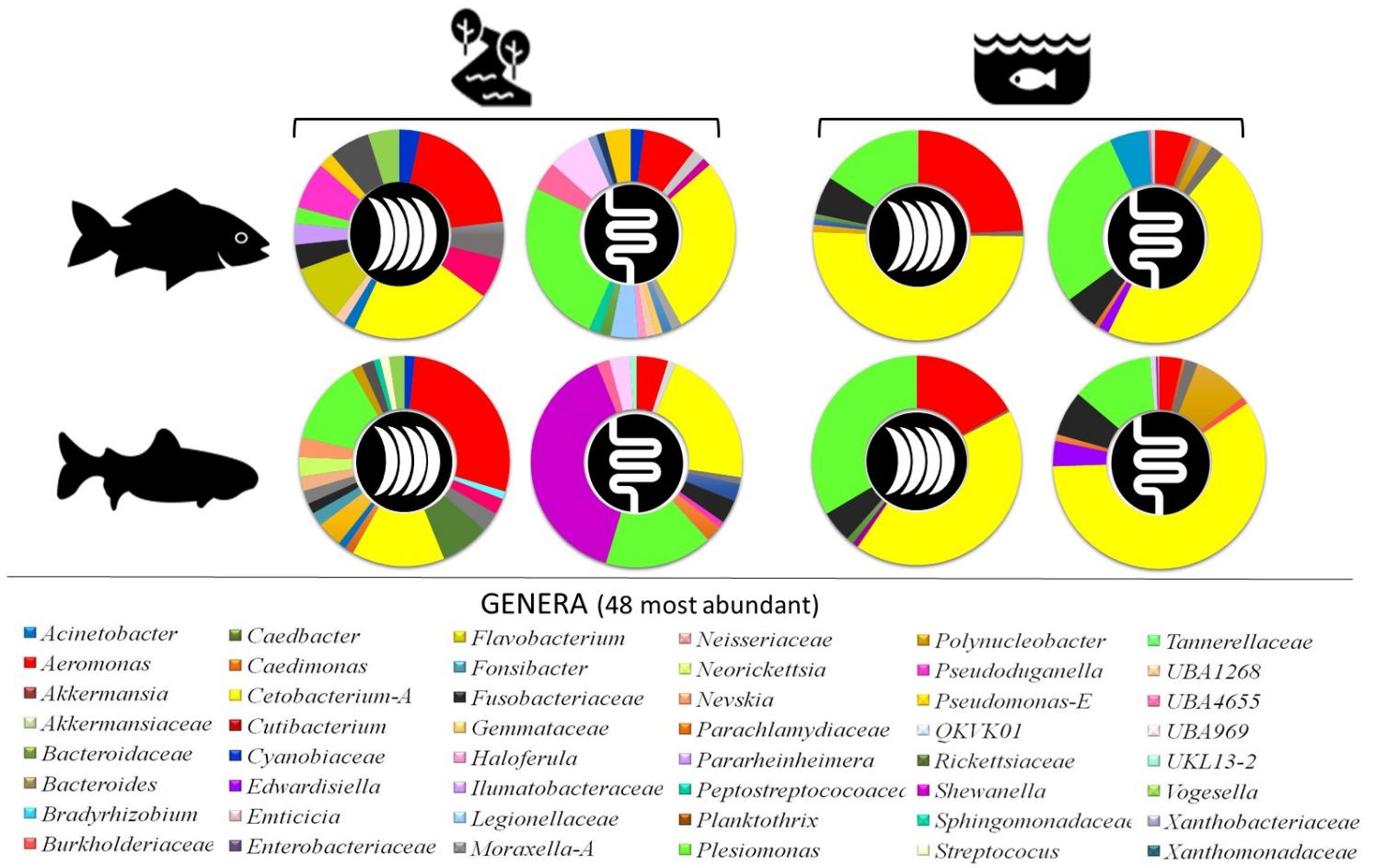


**Supplementary Figure 2.** Comparison of the taxonomic composition of the gills and intestinal microbiomes of *R. aspera* and *P. argenteus* collected in SFR and SPI, highlighting the bacterial phylotypes present in average relative abundance (ARM (≥1%) at gender level).

**Supplementary Table 1** - Parameters of water quality in the period of fish capture in disagreement with the legal limits established COPAM/CERH-MG/2008.

| Parameters (IGAM) | SFR (SF9) | COPAM/CERH-MG/2008 |
| --- | --- | --- |
| Total lead | 0.012 mg/L | 0.01mg/L |
| Total phosphorous | 0.14 mg/L | 0.1 mg/L |
| Total manganese | 0.1701 mg/L | 001mg/L |
| Optical density | 4.4 mg/L | >5 mg/L |
| Cyanobacteria* | 56.209,14 cel/mL | 50.000 cel/mL |
| Mycrocistin | 1.29μg/L | < 1μg/L |

Joint Normative Deliberation COPAM/CERH-MG No. 1, of May 5, 2008, for waters classified in class 2. IGAM (Mineiro Institute for Water Management). SF9 - Water Sampling Station where the animals were collected. *Species of cyanobacteria (*Microcystis* sp. and *Pseudoanabaena* *mucicola*).

**Supplementary Table 2** – Diversity indices analyses.

|  | **Shannon** | | | | **Simpson** | | | |
| --- | --- | --- | --- | --- | --- | --- | --- | --- |
| **Treatment** | **Sum Sq** | **Mean Sq** | **F value** | **Pr(>F)** | **Sum Sq** | **Mean Sq** | **F value** | **Pr(>F)** |
| **Environment** | **5.041** | **5.041** | **18.33** | **0.0005728** | **0.1123** | **0.1123** | **8.29** | **0.0109** |
| Tissue | 0.1964 | 0.1964 | 0.7141 | 0.4105 | 0.02808 | 0.02808 | 2.072 | 0.1693 |
| Specie | 0.08816 | 0.08816 | 0.3205 | 0.5791 | 0.003304 | 0.003304 | 0.2439 | 0.6281 |
| Environment : Tissue | 0.5186 | 0.5186 | 1.885 | 0.1887 | 0.007028 | 0.007028 | 0.5187 | 0.4818 |
| Environment : Specie | 0.451 | 0.451 | 1.64 | 0.2186 | 0.003123 | 0.003123 | 0.2305 | 0.6376 |
| Tissue : Specie | 0.1664 | 0.1664 | 0.6048 | 0.4481 | 2.704e-05 | 2.704e-05 | 0.001996 | 0.9649 |
| Environment : Tissue : Specie | 0.4001 | 0.4001 | 1.455 | 0.2453 | 0.02729 | 0.02729 | 2.014 | 0.175 |

**Supplementary Table 3** – Taxonomic Classification (file attached separately)
